# Impostor Phenomenon and L2 willingness to communicate: Testing communication anxiety and perceived L2 competence as mediators

**DOI:** 10.3389/fpsyg.2022.1060091

**Published:** 2023-01-09

**Authors:** Kay Brauer, Elyas Barabadi, Elham Aghaee, Fakieh Alrabai, Majid Elahi Shirvan, Rebekka Sendatzki, Linnea Marie Vierow

**Affiliations:** ^1^Department of Psychology, Martin Luther University Halle-Wittenberg, Halle (Saale), Germany; ^2^Department of Foreign Languages, University of Bojnord, Bojnord, Iran; ^3^Department of English, Faculty of Languages and Translation, King Khalid University, Abha, Saudi Arabia

**Keywords:** impostor phenomenon, second language, willingness to communicate, anxiety, competence, communication anxiety

## Abstract

The Impostor Phenomenon (IP) describes experiences of perceived intellectual fraudulence despite the existence of objectively good performances, and it is a robust predictor of experiences and outcomes in higher education. We examined the role of the IP in the domain of second language (L2) acquisition by testing its relations with a robust predictor of L2 use, willingness to communicate (WTC). We collected self-reports of 400 adult Iranian L2 learners and tested the associations between the IP and WTC. As expected, we found a negative association between IP and WTC (*r* = −0.13). When testing a mediation model with perceived competence and communication anxiety as parallel mediators, we found evidence for full mediation *via* perceived competence. Our findings show the importance of considering self-evaluations in the domain of L2 acquisition. Further implications and limitations are discussed.

## 1. Introduction

The Impostor Phenomenon (IP; Clance, 1985) describes individual differences in feelings of intellectual fraudulence despite the existence of objective positive performance feedback (e.g., grades and recommendation letters). Those with high expressions in IP (“Impostors”) constantly dismiss positive evaluations of their performance. Foremost, by attributing their success to chance and luck instead of their ability (e.g., Brauer and Proyer, 2022). The IP relates to low engagement in striving for opportunities to advance their career and educational achievements (e.g., Neureiter and Traut-Mattausch, 2016, 2017; Parkman, 2016; Cisco, 2020). We aimed to extend the knowledge on the IP in the educational domain by investigating its role in second-language acquisition (L2) among adult learners. IP-typical self-perceptions of inability might hinder the process of learning a second language, as the latter requires learners to engage in active learning, experience progress throughout their learning process, and have positive experiences when utilizing their skill set. We expected that the IP is negatively associated with L2 learning because the IP robustly relates to experiences of underachievement, irrespective of *actual* achievements (Sakulku and Alexander, 2011). To address this, we collected data from Iranian L2 learners and tested the association between the IP and willingness to communicate (WTC), an immediate predictor of L2 use (MacIntyre et al., 1998). Moreover, we considered indirect effects of communication anxiety and perceived competence on the association between the IP and WTC. To our knowledge, no study has hitherto examined the role of the IP for WTC in L2, and we aimed to narrow this gap in the literature to extend the understanding of self-perceptions of intellectual fraudulence in the domain of L2 acquisition.

### 1.1. The Impostor Phenomenon

Clance (1985) described the IP as an inclination to underestimate one’s own abilities and fear of being exposed as an intellectual fraud. Impostors are convinced that they are “intellectual phonies,” and despite the existence of objective indicators of their successes, such as grades or recommendation letters, they discount their positive performance feedback and assume that they would not be able to repeat their successes (Clance, 1985). The IP has been studied regarding its underlying mechanisms and consequences. Foremost, the IP-typical attributional style explains how Impostors perceive the causes of their successes. Impostors show external-instable-specific attributions of positive performance outcomes, while attributions of events in social contexts and negative events are unrelated to the IP (e.g., Brauer and Wolf, 2016). Studies analyzing self-reports, vignettes, and experiments showed that the IP robustly relates to externalizing success and experiencing negative emotions after positive feedback (e.g., Thompson et al., 1998; Brauer and Wolf, 2016; Badawy et al., 2018; Vaughn et al., 2020; Brauer and Proyer, 2022). Thus, Impostors discount their ability by attributing their positive performances and successes externally to chance and luck. Finally, Impostors’ attributions are biased because their actual performance is unrelated to the IP (e.g., Cozzarelli and Major, 1990; Brauer and Proyer, 2022).

The IP is unrelated to age, study fields, and vocations (Sakulku and Alexander, 2011). Findings on gender differences are mixed, with some studies showing that women experience on average higher levels of IP than men, but effect sizes are small (e.g., Chrisman et al., 1995; Brauer and Wolf, 2016; Badawy et al., 2018). Comparisons between students and working professionals showed that the IP is more pronounced among university students, with robust effects of medium size (Hedges’ *g*s ≈ 0.50; Brauer and Proyer, 2017, 2019; Neureiter and Traut-Mattausch, 2016). The IP relates to numerous negative consequences concerning mental health (e.g., greater levels of anxiety, depressiveness, and neuroticism; e.g., Sakulku and Alexander, 2011; Vergauwe et al., 2015) and one’s career. For example, Impostors show less inclinations to career planning and-striving, less motivation to lead, lower occupational self-efficacy, fewer resources for adapting in their careers, and lower job satisfaction to name but a few (Vergauwe et al., 2015; Neureiter and Traut-Mattausch, 2016, 2017).

### 1.2. The Impostor Phenomenon in education

The IP relates negatively to students’ academic self-evaluations and achievement orientations (e.g., King and Cooley, 1995; Parkman, 2016; Cisco, 2020) and goes along with lower self-efficacy, self-esteem, growth mindset, and performance-efficacy (e.g., Cozzarelli and Major, 1990; Chrisman et al., 1995; Brauer and Wolf, 2016; Bravata et al., 2020; Fleischhauer et al., 2021). Further, the IP shows positive associations with students’ anxiety (i.e., generalized and test anxiety; e.g., Clance, 1985; Chrisman et al., 1995; Thompson et al., 1998), depressiveness, student status stress, psychological distress, and learned helplessness (e.g., Clance, 1985; Chrisman et al., 1995; Thompson et al., 1998; Brauer and Wolf, 2016; Ibrahim et al., 2022; see also Sakulku and Alexander, 2011). Thus, the IP goes along with negative experiences in the educational context.

Despite the well-acknowledged negative links between the IP and learning in different domains (e.g., Blondeau and Awad, 2018; Lee et al., 2022), there is yet limited knowledge on the IP and its role in learning a second/foreign language. Yamini and Mandanizadeh (2011) found that the IP relates negatively to self-efficacy concerning L2 writing abilities in 94 Iranian learners of English as L2. Expanding on their research, we anticipated that language learners with inclinations to the IP would be more prone to experience language anxiety in terms of communication anxiety, fear of negative evaluation, and test anxiety. Based on the well-documented negative association between language anxiety and learners’ WTC in the L2 (e.g., Elahi Shirvan et al., 2019; Dewaele and Dewaele, 2020; Alrabai, 2022), we anticipated the IP to relate negatively to students’ WTC in the target foreign language.

### 1.3. The present study

We aimed to extend the knowledge on the role of the IP among L2 learners. As a criterion, we examined WTC, which describes “the intention to speak or to remain silent, given free choice” and “a readiness to enter into discourse at a particular time with a specific person or persons, using a L2” (MacIntyre et al., 1998, p. 547). WTC relates robustly to self-evaluations, teacher-ratings, and objective indicators of L2 proficiency and is considered the most immediate predictor of L2 use (e.g., MacIntyre et al., 1998; Barabadi et al., 2021). In accordance with the literature, we treat WTC as an indicator of inclinations to engage in L2, which is important for the learning process of a foreign language. Speaking a foreign language during the learning process can expose L2 learners’ grammatical and vocal expressions of the new language and go along with experiences of shame and anxiety (Teimouri, 2018; Wilson and Lewandowska-Tomaszczyk, 2019). We expected that the IP relates negatively to WTC (H1), considering that Impostors’ academic self-concept, which is characterized by, for example, low academic self-esteem, self-efficacy, and discount of abilities, translates into being less inclined to expose their performance (i.e., speaking the foreign language) in front of others such as teachers and co-students.

Additionally, we examined the role of communication anxiety and self-perceived competence as mediator variables. Communication anxiety describes “worry and negative emotional reaction aroused when learning or using a second language” (MacIntyre, 1999, p. 27). According to Bravata et al. (2020), anxiety is deeply linked to the IP in that it is typically presumed to precede and trigger impostor feelings among young people and adults alike. Also, its components have been well studied in relation to the IP. For example, fear of negative evaluation is one significant aspect of anxiety, and Chrisman et al. (1995) found that Impostors usually report greater levels of fear of being negatively evaluated by others. It seems that in the context of learning, students who experience certain levels of IP are likely to demonstrate a higher tendency for fear of negative evaluation by their teachers or peers. Accordingly, we assessed communication anxiety as a potential mediator variable to examine its indirect effect on the IP—WTC association. We expected a positive association between the IP and communication anxiety (H2a).

**Figure 1:**
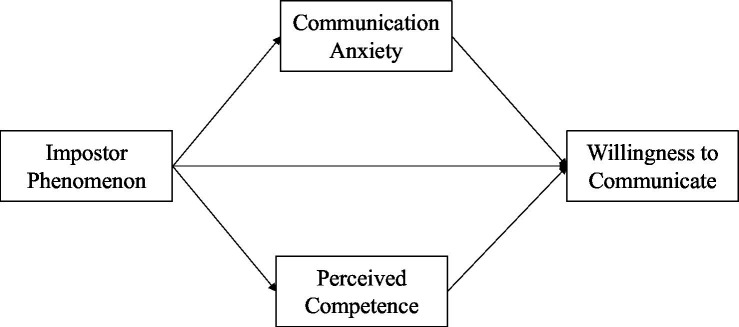
Parallel mediation model testing the association between the Impostor Phenomenon and willingness to communicate under consideration of perceived competence and communication anxiety.

MacIntyre and Charos (1996) suggested that L2 learners’ self-assessment of their L2 competence might be more important for their L2 communication than their actual L2 ability. Thus, self-perceptions of competence play a role in learners’ inclinations to L2 use. There is robust evidence that the IP is characterized by low academic self-evaluations, academic self-esteem, and inclinations to underestimate their abilities (e.g., Cozzarelli and Major, 1990; Thompson et al., 1998; Badawy et al., 2018; Brauer and Proyer, 2022), and that self-perceived competence relates positively to WTC (e.g., Dewaele, 2008; Piechurska-Kuciel, 2011; Lockley, 2013). Further, Yamini and Mandanizadeh (2011) showed that the IP relates negatively to self-efficacy in L2 abilities. Therefore, we also took self-perceived competence as a potential mediator variable into account and expected that the IP relates negatively to self-evaluations of competence (H2b). Using a parallel mediation analysis model, we examined whether the hypothesized negative association between the IP and WTC (expected in H1) would be explained by indirect effects of anxiety and self-perceived competence (see Figure 1 for the model).

## 2. Materials and method

### 2.1. Sample and procedure

Our sample comprised *N* = 400 (15% male and 84% female) participants who studied Teaching English as a Foreign Language (TEFL). The majority (96.5%) were BA students and the remainder were master’s or PhD students. Since most participants are BA students of TEFL, we present the main courses that they are required to take during their BA program: four language skills, grammar, study skills, linguistics, research, testing, teaching methodology, material development, and a practicum. Overall, BA students must complete 136 credit courses during the course of eight semesters. For each “two-credit” course, students have to attend 16 sessions. Participants’ mean age was 21.1 years (*SD* = 4.1; median = 20). We collected all data online using GoogleDoc. At the time of data collection, all university classes were held online during the COVID-19 pandemic. We recruited our participants from three Iranian universities (Ferdowsi University of Mashhad, University of Tehran, and University of Bojnord) and shared the link to the online questionnaire in L2 classes. Institutional approval is not required for this type of research at the authors’ universities. Participation was anonymous, with informed consent, and in accordance with the 1964 Declaration of Helsinki.

### 2.2. Instruments

We used self-report questionnaires to assess the study variables. Participants gave their responses to the items on 5-point Likert-type scales (1 = *strongly disagree*; 5 = *strongly agree*).

#### 2.2.1. Impostor Phenomenon

We used the *Clance Impostor Phenomenon Scale* (CIPS; Clance, 1985) for the assessment of the IP in its Persian translation. The CIPS comprises 20 items (e.g., “When people praise me for an achievement, I fear that I will not be able to meet their expectations in the future.”). There is good evidence for the reliability and validity of the instrument in different language versions (for an overview, see Mak et al., 2019), and we found good internal consistency in our study (α = 0.84).

#### 2.2.2. Willingness to communicate

We assessed willingness to communicate (WTC) with seven items that were originally developed by Weaver (2005) and adapted by Khajavy et al. (2018) for their use in the context of foreign language learning. A sample item is “I am willing to speak English about a topic which is written on a board or a piece of paper.” In our study, the internal consistency was high (α = 0.87).

#### 2.2.3. Communication anxiety

We used Dewaele and Dewaele’s (2020) 8-item scale to assess L2 communication anxiety. This scale assesses the extent to which L2 learners experience L2 communication anxiety when speaking English as foreign language. A sample item is “I get nervous and confused when I am speaking in my FL class.” The results of the current study provided evidence for the reliability and validity of this scale. The internal consistency was high (α = 0.87) in our study.

#### 2.2.4. Perceived L2 competence

We assessed perceived L2 competence (PC) with eight items from Peng and Woodrow (2010) and Fushino (2010). This scale assesses the extent to which L2 learners perceive their competence in L2. A sample item is “I am able to do a role-play standing in front of the class in English (e.g., ordering food in a restaurant).” The internal consistency was α = 0.91.

### 2.3. Data analysis

We examined the intercorrelations between our study variables by computing bivariate correlations for preliminary analyses. Power analyses with G*Power (Faul et al., 2007) showed that our sample size allowed to detect minor effects (*ρ* = 0.16) with 90% power and 5% type I error-rate (two-tailed).

We tested our hypotheses with regression analyses in *Mplus* 8.6 (Muthén and Muthén, 1997/2017): First, we tested the baseline model with IP as the independent variable and WTC as outcome. Secondly, we tested our parallel mediation model, which included anxiety and PC as mediator variables for the association between IP and WTC (see Figure 1 for a visualization of the model). We used maximum likelihood estimation and report unstandardized path coefficients (*b*), standard errors (SE), 95% confidence intervals (CI), *p*-values, and the determination coefficient *R*^2^. We computed bootstrapped 95% CIs (*k* = 5,000 samples) for all parameters. In line with MacKinnon et al. (2007), we tested the statistical significance of the indirect effects^1^ on basis of the 95% CIs. Accordingly, the existence of an indirect effect is supported when the CI does not include zero (MacKinnon et al., 2007). In all analyses, we controlled for gender as a covariate to account for potential gender differences as described in the literature (e.g., Badawy et al., 2018; Fleischhauer et al., 2021).

## 3. Results

### 3.1. Preliminary analyses

The descriptive statistics are displayed in Table 1. The means and *SD*s of the scale scores were comparable to prior studies (e.g., Brauer and Wolf, 2016; Barabadi et al., 2021). The kurtosis (≤ 0.92) and skewness (≤ 0.58) of the study variables did not indicate deviations from normality. Associations with gender were negligible (*r*s ≤ 0.05, *p*s ≥ 0.153), but women showed a tendency to higher WTC (*r* = −0.12, *p* = 0.016). Age was unrelated to all study variables (*r*s ≤ 0.10).

**Table 1:** Descriptive statistics and correlations of the Impostor Phenomenon (IP), anxiety, perceived competence (PC), and willingness to communicate (WTC).

	Descriptive statistics		Inter-correlations			
	*M*	*SD*	IP	Anxiety	PC	WTC
IP	54.16	11.74	-	0.45***	−0.14**	−0.11*
Anxiety	2.82	1.01	0.45***	-	−0.61***	−0.44***
PC	3.87	0.78	−0.15**	−0.61***	-	0.62***
WTC	3.87	0.75	−0.12**	−0.45***	0.62***	-

*N* = 400. Above diagonal zero-order correlations and below diagonal second-order correlations controlled for age and gender. *p < 0.05. **p < 0.01. ***p < 0.001. Two-tailed. Harman’s one-factor test showed that 25.9% of the variance in the study variables were explained by a single factor, which is below the cut-off (50%) suggesting the existence of robust common method variance.

The correlations among the study variables were as expected, with a positive association between the IP and anxiety (*r* = 0.45), a positive association between PC and WTC (*r* = 0.62), as well as negative correlations between anxiety and PC (*r* = −0.61) and WTC (*r* = −0.45; all coefficients controlled for gender; Table 1). In line with H1, IP related negatively to WTC (*r* = −0.12, *p* = 0.014), but the effect was small. As hypothesized, the IP related positively to communication anxiety (*r* = 0.45, *p* < 0.001; H2a), whereas higher IP did go along with perceiving oneself as low in competence (*r* = −0.15, *p* = 0.003; H2b).

### 3.2. Regression analyses

The baseline model showed the expected negative association between the IP and WTC (β = −0.12, *p* = 0.015; Table 2), but the effect size was small. The model explained 2.9% of the variance in WTC. Thus, we found support for H1.

**Table 2:** Parallel mediation analyzes testing indirect effects of anxiety and perceived competence (PC) on the association between Impostor Phenomenon (IP) and willingness to communicate (WTC).

	*b*	SE	95% CI	*p*
Baseline model (*R*^2^ = 0.029)				
IP ➔ WTC	**−0.01**	0.003	[−0.014, −0.001]	0.017
Mediation model (*R*^2^ = 0.398)				
Direct paths				
IP ➔ WTC	0.00	0.003	[−0.006, 0.007]	0.867
IP ➔ Anxiety	**0.04**	0.004	[0.030, 0.048]	<0.001
IP ➔ PC	**−0.01**	0.003	[−0.017, −0.003]	0.003
Anxiety ➔ WTC	−0.08	0.046	[−0.175, 0.005]	0.066
PC ➔ WTC	**0.52**	0.059	[0.407, 0.638]	<0.001
Indirect paths				
IP ➔ Anxiety ➔ WTC	0.00	0.002	[−0.007, 0.000]	0.078
IP ➔ PC ➔ WTC	**−0.01**	0.002	[−0.009, −0.002]	0.008
Total indirect effect	**−0.01**	0.003	[−0.014, −0.004]	0.001
Total effect	**−0.01**	0.003	[−0.014, −0.001]	0.017

*N* = 400. Bold coefficients: bootstrapped 95% CI (*k* = 5,000 samples) excludes zero. The *χ*^2^ model test indicated that the mediation model was superior to a null model assuming no associations among the study variables (*χ*^2^ = 498.09, df = 9, *p* < 0.001). The covariate gender had an effect of *b* = −0.26 (*β* = −0.13, *p* = 0.011) in the baseline model and *b* = −0.20 (95% CI [−0.343, −0.055], SE = 0.073, *p* = 0.007) in the mediation model.

Next, we tested the mediation model with communication anxiety and perceived competence as mediator variables (see Figure 1; Table 2). The direct path between the IP and WTC became negligible and non-significant (*b* = 0.00, 95% CI [−0.006, 0.007]) after adding anxiety and PC. We found that anxiety and PC yielded a statistically significant negative total indirect effect (*b* = −0.01, 95% CI [−0.014, −0.004]) on the association between IP and WTC. The inspection of the single indirect effects showed an indirect effect of PC (*b* = −0.01, 95% CI [−0.009, −0.002]), whereas anxiety did not explain the IP—WTC relationship (*b* = 0.00, 95% CI [−0.007, 0.000]). Thus, higher IP related to lower self-perceptions of competence and thereby less WTC. The mediation model explained 39.8% of the variance in WTC. In summary, the IP—WTC association was fully mediated *via* PC (Muthén et al., 2017).

## 4. Discussion

Our study aimed to extend the knowledge regarding the IP in the educational domain by examining its role in L2 learners. We tested the relationship between the IP and WTC, a robust predictor of L2 use (MacIntyre et al., 1998). As expected, our data showed that the IP goes along with lower WTC among L2 learners. However, the effect size was small, suggesting that the IP might play only a minor role in L2 learning when evaluating the direct effect. We additionally assessed whether communication anxiety and perceived competence might yield indirect effects on the IP—WTC association. We found that the IP—WTC association was mediated by the indirect effect *via* perceived competence, whereas the contribution of anxiety was negligible. Perceived competence has been identified as a robust predictor of WTC in prior studies (e.g., Dewaele, 2008; Piechurska-Kuciel, 2011; Lockley, 2013), and this aligns well with Impostors’ tendency to discount their abilities and underestimate their competence (e.g., Brauer and Proyer, 2022). This is also consistent with Yamini and Mandanizadeh’s (2011) finding that the IP is associated with lower self-efficacy and, as a result, lower self-perceived writing competencies in L2. Our findings show that the IP’s negative association with PC robustly relates to WTC and that Impostors’ inclinations to dismiss their competencies is detrimental to engaging in communicating in the foreign language. Thus, while the IP plays only a minor role in terms of its *direct* effect on WTC, Impostors’ tendencies to perceive themselves as low in competence contribute *indirectly* to understanding inhibitions in WTC in L2 learners. One might argue that there might be redundancy between the IP and PC, but our correlation analyzes showed that the expected overlap was far from redundancy, with less than 2.3% shared variance.

Although our expectations concerning the direction of the associations between communication anxiety and the IP and WTC were met in line with prior findings (e.g., Chrisman et al., 1995; Barabadi et al., 2021), the indirect effect of communication anxiety on the IP—WTC association did not reach statistical significance. Thus, although anxiety robustly related to the IP and WTC with comparatively strong correlation effects, its mediating role for the IP—WTC relationship was negligible. It could be argued that although our expectations concerning the directions of the effects were met for the relations between IP and anxiety, and anxiety and WTC, their joint effect was negligible when perceived competence is considered in the model. It is desirable that future research clarifies the statistical robustness and practical relevance of this finding, as well as extending research on anxiety for L2 learning (e.g., by considering fine-grained antecedents of anxiety such as shame; Teimouri, 2018; Wilson and Lewandowska-Tomaszczyk, 2019). Finally, one might argue that Impostors are more affected by discounting their positive potential (i.e., competence) than by negative emotions such as anxiety (e.g., Yamini and Mandanizadeh, 2011; Brauer and Proyer, 2022).

In conclusion, the IP showed a minor effect size in relation to WTC, which is fully mediated by Impostors’ inclinations to experience low competencies when it comes to their ability to speak a foreign language. Our findings have several implications. While Impostors’ dismissal of their abilities has been evaluated with regard to indicators of generalized abilities in terms of intelligence or grades (e.g., Cozzarelli and Major, 1990; Brauer and Proyer, 2022), our findings highlight that the IP and its tendency to discount success, performance, and competencies also affect domains such as WTC in the context of learning a foreign language. We argue that identifying the discounting of competencies as a robust mediator contributes to understanding the previously documented consequences of the IP, as well as providing an avenue for future research and the development of trainings and interventions to reduce the IP. Considering that there is increasing evidence showing that the dismissal of abilities is a core criterion of the IP, future studies could use this knowledge to examine the efficacy of trainings. For example, Proudfoot et al. (2009) examined an intervention program among workers that increased “self-serving” attributional styles and showed positive effects on outcomes such as well-being and job satisfaction. An intervention aimed at training to internalize positive performance feedback from teachers and peers, as well as formal feedback (e.g., grades), might help to reduce the IP. Interventions could also increase Impostors’ self-perceptions of competence, academic self-esteem, and self-efficacy to a more veridical level, and support them in achieving goals such as learning a foreign language, irrespective of personal or professional motivations. Considering the role of the IP and its mental health correlates, we would expect such training to help alleviate the effects on outcomes such as anxiety (including its fine-grained components such as communication-and test anxiety), fear of negative evaluation, and depressiveness (e.g., Chrisman et al., 1995; Yamini and Mandanizadeh, 2011; Brauer and Wolf, 2016; Bravata et al., 2020).

### 4.1. Limitations and future directions

Our findings must be interpreted with several limitations. Although there is robust evidence that the IP longitudinally predicts perceptions of competence (e.g., Cozzarelli and Major, 1990; Brauer and Proyer, 2022) and that the IP precedes WTC from a theoretical perspective, our findings must be interpreted with caution because longitudinal research is needed to examine the causal pathways between the study variables in the narrow sense, especially for the indirect effect identified in our mediation model. Secondly, we only collected data from Iranian L2 learners as part of TEFL, and replication in other countries and alternative L2 target languages are important to generalize our findings. Thirdly, our findings are based on self-reports and should be extended by supplementing self-reports of WTC by L2 abilities assessed with standardized tests and teacher evaluations to reduce IP-typical biases and common method variance (Campbell and Fiske, 1959). Fourth, our sample is not representative, as it comprises comparatively young participants of high educational status from Iran, which limits the generalizability of our findings. Finally, we collected the data during the COVID-19 pandemic, which has affected teaching and learning (e.g., by illness or loneliness during isolation).

Future research could extend our findings in multiple ways. Although WTC is a robust predictor of L2 use, we have not assessed external indicators of L2 use. For example, it would be interesting to examine the associations between the IP and objective indicators of L2 acquisition such as test-and exam data and incorporate teacher ratings of students’ L2 proficiency. While it is well-supported that WTC robustly relates to objective indicators such as tests or teacher ratings (e.g., Barabadi et al., 2021), using different sources of information would also allow to examine the discrepancies between Impostors’ self-perceived competencies and external evaluations of their abilities. For example, initial research testing Impostors’ self-evaluations in creativity in comparison to their results in a situational judgment test of creative styles has shown that the IP goes along with discrepancies between self-evaluations and test data, indicating that they underestimate their creative abilities (Proyer and Brauer, 2020). Similarly, we expect that Impostors would systematically underestimate their L2 abilities in comparison to an external criterion (e.g., teacher evaluations or language-test scores).

Further, future studies should extend the nomological net of the IP in the domain of L2 learning. One could argue that the IP would negatively correlate with other concepts relating to WTC such as communication confidence (Lee and Drajati, 2019; Lee and Hsieh, 2019) and motivation (Khajavy et al., 2016; Lee and Drajati, 2019). In this regard, it is likely that the IP would positively correlate with emotions that negatively affect learners’ WTC, like generalized anxiety and boredom (e.g., Pawlak et al., 2016) and negatively with emotions that positively influence WTC in L2 such as enjoyment (Teimouri, 2018; Dewaele and Dewaele, 2020; Alrabai, 2022). This line of research could help in assessing how the IP affects different aspects of language learning and in building an overarching theoretical framework that supports guiding research on the IP in the educational context.

## Data availability statement

The datasets presented in this study can be found in the Open Science Framework under https://osf.io/kyg5c/.

## Ethics statement

Ethical review and approval was not required for the study on human participants in accordance with the local legislation and institutional requirements. The patients/participants provided their written informed consent to participate in this study.

## Author contributions

KB and EB: conceptualization. EB, EA, FA, and MS: data collection. KB and RS: formal analysis. KB, EB, and RS: roles/writing – original draft. KB, EB, EA, FA, MS, RS, and LV: writing – review and editing. All authors contributed to the article and approved the submitted version.

## Conflict of interest

The authors declare that the research was conducted in the absence of any commercial or financial relationships that could be construed as a potential conflict of interest.

## Publisher’s note

All claims expressed in this article are solely those of the authors and do not necessarily represent those of their affiliated organizations, or those of the publisher, the editors and the reviewers. Any product that may be evaluated in this article, or claim that may be made by its manufacturer, is not guaranteed or endorsed by the publisher.

## Footnotes

^1^Note that we use the term "effect" in accordance with the literature. Since our data are cross-sectional, we cannot draw conclusions on effects in the sense of causality.
